# One-Year Comparative Outcomes of Conventional Versus Accelerated Corneal Cross-Linking in Progressive Keratoconus

**DOI:** 10.3390/medicina62061209

**Published:** 2026-06-22

**Authors:** Iva Bešlić, Sania Vidas Pauk, Martina Tomić, Miro Kalauz, Tomislav Kuzman, Sonja Jandroković, Ivan Škegro, Antonela Geber, Lorena Karla Šklebar, Dina Lešin Gaćina, Petar Bešlić, Sanja Masnec

**Affiliations:** 1 Department of Ophthalmology, University Hospital Centre Zagreb, 10000 Zagreb, Croatia; iva.lukac@gmail.com (I.B.); sania_vidas@yahoo.com (S.V.P.); tomislav.kuzman@kbc-zagreb.hr (T.K.); sonja.jandrokovic@kbc-zagreb.hr (S.J.); ivanskegro@yahoo.com (I.Š.); antonela.geber@kbc-zagreb.hr (A.G.); lkskleba@kbc-zagreb.hr (L.K.Š.); dina.lesin@kbc-zagreb.hr (D.L.G.); sanjamp@yahoo.com (S.M.); 2 Department of Ophthalmology, Vuk Vrhovac University Clinic for Diabetes, Endocrinology and Metabolic Diseases, Merkur University Hospital, Dugi dol 4a, 10000 Zagreb, Croatia; martina.tomic@kb-merkur.hr; 3 School of Medicine, University of Zagreb, Šalata 3, 10000 Zagreb, Croatia; 4 Department of Cardiology, Clinical Hospital Sveti Duh, 10000 Zagreb, Croatia; petar.beslic@hotmail.com

**Keywords:** keratoconus, corneal cross-linking, conventional cross-linking, accelerated cross-linking, ABCD classification, Scheimpflug tomography, longitudinal study

## Abstract

*Background and Objectives*: Corneal collagen cross-linking (CXL) halts keratoconus progression, yet potential differences between conventional and accelerated protocols at one year remain uncertain. We analyzed the completed 12-month follow-up of a previously reported 6-month cohort to compare conventional (3 mW/cm^2^ × 30 min; CXL 30) versus accelerated (9 mW/cm^2^ × 10 min; CXL 10) CXL, interpreting outcomes within the ABCD framework alongside Kmax and curvature radii. *Materials and Methods*: In this single-center retrospective longitudinal analysis of prospectively collected routine clinical data, 22 eyes were included, with assessments performed at baseline and at 1, 3, 6, and 12 months of follow-up. Evaluated outcomes comprised ABCD stages (A–D), anterior and posterior radius of curvature (ARC and PRC), Kmax, pachymetric and elevation indices, as well as UDVA and BCVA. Within-group change used Friedman with Wilcoxon post hoc; between-group differences used Mann–Whitney (α = 0.05). *Results*: Both protocols resulted in significant visual improvement and Kmax reduction at 12 months (overall time effect: CXL 30 *p* < 0.001; CXL 10 *p* = 0.026). Median Kmax decreased 56.5 → 52.3 D (CXL 30) and 59.3 → 58.3 D (CXL 10). UDVA improved 0.2 → 0.6 (CXL 30) and 0.2 → 0.3 (CXL 10); BCVA 0.4 → 0.8 (CXL 30) and 0.2 → 0.5 (CXL 10). Tomographic analysis showed predominantly anterior changes, with a significant decrease in A stage in the CXL 30 group and an increase in ARC in both groups, more pronounced in CXL 30. In the late 6 → 12-month window, posterior metrics (PRC and posterior elevation) were largely stable; raw PRC change did not reach significance. *Conclusions*: Conventional and accelerated CXL both stabilized keratoconus at one year with meaningful functional gains. Beyond 6 months, remodeling was predominantly anterior; within-group findings suggested a more pronounced anterior tomographic response in the CXL 30 group. The 12-month visit may be useful for reassessing stability after CXL, although this study was not designed to determine optimal retreatment timing or optical rehabilitation strategy. Longer-term studies with standardized biomechanical and densitometric endpoints are warranted to assess durability and refine protocol selection.

## 1. Introduction

Keratoconus (KC) is a progressive, non-inflammatory ectatic corneal disorder characterized by stromal thinning, anterior protrusion, and irregular astigmatism, which may lead to substantial visual impairment if left untreated [1,2]. Recent reviews have also highlighted marked geographic differences in keratoconus prevalence and the growing role of modern tomography in earlier detection [3,4]. In addition to its clinical effects, keratoconus can substantially affect patients’ quality of life, which further supports timely intervention [5]. Early recognition and treatment are important for preserving visual function and delaying or avoiding keratoplasty. Corneal collagen cross-linking (CXL) has become an established treatment for halting disease progression, and the conventional Dresden epithelium-off (epi-off) protocol (CXL 30: 3 mW/cm^2^ for 30 min; ~5.4 J/cm^2^) has been extensively validated in clinical practice [6,7,8].

Accelerated epi-off regimens, such as 9 mW/cm^2^ for 10 min, were introduced to shorten the procedure time while maintaining the total fluence [9]. This approach is based on Bunsen–Roscoe reciprocity, although riboflavin–UVA photochemistry is also constrained by oxygen diffusion, meaning that irradiance–time trade-offs can influence the depth and footprint of cross-link formation [10,11,12]. Experimental and clinical data suggest that these oxygen-dependent kinetics, together with protocol differences, may affect the depth and pattern of subsequent corneal remodeling [13]. These mechanisms may contribute to differences in the depth or pattern of postoperative corneal change between protocols, although clinical evidence remains heterogeneous. Registry data add an important real-world perspective by showing stability and retreatment patterns over time, with five-year comparisons of standard and accelerated epi-off CXL supporting durable stabilization [14]. However, these studies did not use the ABCD classification as a primary outcome measure [9,14,15,16].

A robust assessment of structural change requires metrics that go beyond single-parameter keratometry and also capture corneal geometry and thickness. The Belin ABCD framework integrates anterior curvature, posterior curvature, thinnest pachymetry, and distance visual acuity, all referenced to the thinnest corneal point, and can be interpreted together with continuous tomographic indices such as anterior and posterior radius of curvature (ARC and PRC), Kmax, pachymetric progression, and elevation-based ectasia metrics [17,18]. This framework helps address some of the limitations of apically based measures, which may under-represent the cone and the posterior surface. Early keratoconus often manifests posterior changes before anterior alteration, while cone location can influence the magnitude and pattern of postoperative flattening [18,19,20].

Several clinical studies have shown that CXL 10 (9 mW/cm^2^ × 10 min) can provide short-term control of progression, anterior remodeling, and visual improvement comparable to CXL 30 [21,22,23]. A smaller number of reports, however, have suggested greater clinical improvement and more pronounced anterior flattening after conventional CXL [24]. Historically, many protocol comparisons focused mainly on anterior-surface keratometry, visual function, and refraction over 6–24 months, and some also used demarcation-line depth at one month as a surrogate of treatment penetration. However, demarcation-line findings have not been fully consistent across techniques and riboflavin formulations [25]. More recent comparative studies have also incorporated densitometry alongside tomographic and functional outcomes, suggesting that protocol-related differences may extend beyond keratometry alone [26,27]. Some of this variability likely reflects differences in protocol design, follow-up duration, and riboflavin formulation across studies. For that reason, standardized multi-parameter tomographic frameworks such as ABCD may be particularly useful when comparing cross-linking protocols.

Although ABCD-based analyses have been increasingly used in studies with about 1 year of follow-up, direct head-to-head comparisons between conventional and accelerated epi-off CXL using this framework remain limited. In particular, there is a lack of longitudinal 12-month comparative data evaluating whether potential differences between protocols become more apparent beyond the early postoperative period.

Therefore, the aim of this study was to analyze the completed 12-month follow-up data from the same clinical cohort and to compare conventional and accelerated CXL using the ABCD classification together with complementary tomographic and functional parameters, with particular attention to whether the short-term similarity observed earlier persisted at one year or whether protocol-related differences in corneal remodeling emerged during the 6 → 12-month interval [28].

## 2. Materials and Methods

Study Design and Ethics. This was a single-center retrospective longitudinal analysis of prospectively collected routine clinical data from the same comparative observational cohort of patients with progressive keratoconus whose 6-month outcomes were previously reported [28]. The present analysis was conducted in accordance with the Declaration of Helsinki, and ethics approval for the present research project was obtained from the Ethics Committee of the University Hospital Centre Zagreb in September 2025 (Class number: 8.1-25/223-2; Reference number: 02/013 AG). Written informed consent for treatment and routine clinical follow-up was obtained. Data were pseudonymized prior to analysis. The study was retrospectively registered at ClinicalTrials.gov in September 2025 (NCT07194538). All examinations were conducted as part of routine clinical care, and the present 12-month analysis was undertaken after completion of follow-up.

Participants and Study Period. Consecutive patients with progressive keratoconus were evaluated and treated between May 2021 and December 2021, and the 12-month follow-up was completed by January 2023. Of the 28 eyes originally included, 22 eyes from 20 consecutive patients completed the 12-month visit and were analyzed in the present per-protocol analysis.

Keratoconus progression was defined according to the Global Consensus on Keratoconus and Ectatic Disease [2]. “Ectasia progression” was defined by a consistent change in at least two of the following parameters, provided that the observed change exceeded the normal measurement noise of the testing system: (1) anterior corneal steepening, (2) posterior corneal steepening, and (3) thinning and/or an increased rate of corneal thickness change from the periphery toward the thinnest point. After progression had been confirmed, patients underwent either conventional (CXL 30: 3 mW/cm^2^ for 30 min) or accelerated (CXL 10: 9 mW/cm^2^ for 10 min) corneal cross-linking at the treating surgeon’s discretion as part of routine care [6,9]. Treatment allocation (CXL 30 or CXL 10) was based on the treating surgeon’s clinical judgment, and no randomization was performed.

Eligibility criteria. Inclusion required progressive keratoconus documented on serial Scheimpflug tomography (Pentacam HR; OCULUS Optikgeräte GmbH, Wetzlar, Germany) within the prior 12 months; a clear corneal axis; and corneal thickness 370 µm or more. Exclusion criteria included central corneal scarring, chemical injury, severe corneal infection, ocular surface disease, pregnancy or lactation during treatment, previous herpetic keratitis, systemic autoimmune disease, and inadequate-quality tomography.

Cross-linking Protocols. All patients underwent epi-off CXL with either the conventional protocol (CXL 30: 3 mW/cm^2^ for 30 min; total fluence 5.4 J/cm^2^) or the accelerated protocol (CXL 10: 9 mW/cm^2^ for 10 min; total fluence 5.4 J/cm^2^). The corneal epithelium was mechanically removed using a blunt hockey knife, after which the cornea was soaked with 0.1% riboflavin in 1.1% hydroxypropyl methylcellulose (MedioCROSS M, Medio-Haus-Medizinprodukte GmbH, Kiel, Germany) for 10 min, with applications every 2 min. Eyes in the CXL 30 group were then exposed to UVA light using the CCL Vario system (Peschke Trade GmbH, Hünenberg, Switzerland) at 3 mW/cm^2^ for 30 min, while those in the CXL 10 group received 9 mW/cm^2^ for 10 min. Riboflavin was reapplied every 5 min throughout UVA exposure. All cross-linking procedures were performed by the second author. At the completion of the procedure, the cornea was rinsed with balanced salt solution, followed by topical administration of a dual-antibiotic regimen and dexamethasone. A bandage soft contact lens was applied and left in place for six days. During the early postoperative period, patients used preservative-free topical moxifloxacin in combination with a tobramycin–dexamethasone combination for up to 6 days. After contact lens removal, tobramycin–dexamethasone was continued with a gradual taper, after which patients transitioned to preservative-free dexamethasone once to twice daily for one month.

Examinations and Outcome Measures. Routine clinical assessments were performed preoperatively (V0) and postoperatively at 1 month (V1), 3 months (V2), 6 months (V3), and 12 months (V4). Assessments included UDVA and BCVA recorded in decimal Snellen notation, slit-lamp biomicroscopy, and Scheimpflug tomography (Pentacam HR; OCULUS Optikgeräte GmbH, Wetzlar, Germany). Approximate logMAR equivalents were additionally provided for key baseline-to-12-month visual acuity outcomes to improve international comparability. All corneal imaging examinations were performed by the second author and subsequently reviewed by the first author.

The Belin ABCD classification was used as the main structural assessment tool, including A and B stages derived from the anterior and posterior radius of curvature measured in a 3.0 mm zone centered on the thinnest corneal point, as well as C (thinnest pachymetry) and D (distance visual acuity) stages (ordinal scale 0–4; lower values indicate less advanced disease). For the present analysis, the ABCD A stage was used as the primary analytical framework, while all ABCD stages, together with complementary tomographic indices, were analyzed to provide a comprehensive assessment of one-year corneal remodeling.

Secondary outcome measures included continuous tomographic indices: anterior and posterior radius of curvature (ARC, PRC; mm, higher = flatter), maximum keratometry (Kmax, D), thinnest pachymetry (TP), front and back mean keratometry (F Km/B Km), anterior and posterior elevation at the thinnest point (El. F, El. B), pachymetry at the apex (PA), Belin/Ambrósio deviation (BAD-D), Ambrosio relational thickness (ARTmax), and pachymetric progression indices (PI).

Statistical Analysis. Statistical analyses and graph preparation were performed using Statistica 14.0.1.25 (TIBCO Software Inc, San Ramon, CA, USA). Continuous variables were reported as mean ± SD or median (min–max), and categorical variables as counts and percentages. Data distribution was assessed with the Shapiro–Wilk test, and parametric or nonparametric methods were applied accordingly. Differences between two independent groups were analyzed using the t-test or Mann–Whitney test, whereas repeated measurements were evaluated using repeated-measures ANOVA or Friedman ANOVA. Post hoc comparisons were performed using the Scheffe or Wilcoxon tests. Categorical variables were compared using the Chi-square test. A *p*-value < 0.05 was considered statistically significant.

## 3. Results

A total of 22 eyes from 20 patients (17 males and 3 females; mean age 25.40 ± 5.53 years) with progressive keratoconus who underwent CXL were included in the present analysis and had completed 12 months of follow-up. Based on treatment duration, eyes were allocated to one of two protocol groups: the conventional CXL 30 group (*n* = 11), which underwent 30 min of irradiation, and the accelerated CXL 10 group (*n* = 11), which underwent 10 min of irradiation.

Baseline characteristics of the patients (eyes) in the two treatment groups are summarized in Table 1. Patients in the CXL 30 group were significantly younger, and the proportion of female patients was higher than in the CXL 10 group (*p* < 0.001). No statistically significant between-group differences were observed for eye laterality, Kmax, visual acuity, or refractive error (*p* > 0.05). Because the primary analyses mainly assessed within-group changes over time, differences in statistical significance between groups were not interpreted as definitive evidence of true between-group differences.

In both treatment groups, median UDVA and BCVA increased significantly over the course of follow-up (UDVA: CXL 30 *p* = 0.012, CXL 10 *p* = 0.021; BCVA: both groups *p* < 0.001), whereas Kmax showed a significant reduction from baseline across study visits in both groups (CXL 30 *p* < 0.001, CXL 10 *p* = 0.026) (Table 2, per-protocol set). UDVA improved from 0.2 to 0.6 in the CXL 30 group and from 0.2 to 0.3 in the CXL 10 group, corresponding approximately to logMAR 0.70 to 0.22 and 0.70 to 0.52, respectively. BCVA improved from 0.4 to 0.8 in the CXL 30 group and from 0.2 to 0.5 in the CXL 10 group, corresponding to logMAR values of approximately 0.40 to 0.10 and 0.70 to 0.30, respectively. Post hoc analysis for UDVA demonstrated a significant improvement from V2 to V3 in the CXL 30 group (Z = 2.2023, *p* = 0.043) and from V1 to V2 in the CXL 10 group (Z = 1.826, *p* = 0.048). For BCVA, a significant increase was observed in both groups between V1 and V2 (CXL 30: Z = 2.240, *p* = 0.025; CXL 10: Z = 2.521, *p* = 0.012), with an additional significant improvement from V2 to V3 in the CXL 10 group (Z = 2.201, *p* = 0.028). In contrast, Kmax decreased significantly between V1 and V2 (Z = 2.701, *p* = 0.007) and between V2 and V3 (Z = 2.395, *p* = 0.017) only in the CXL 30 group, whereas interval changes in the CXL 10 group were not statistically significant.

**Figure 1:**
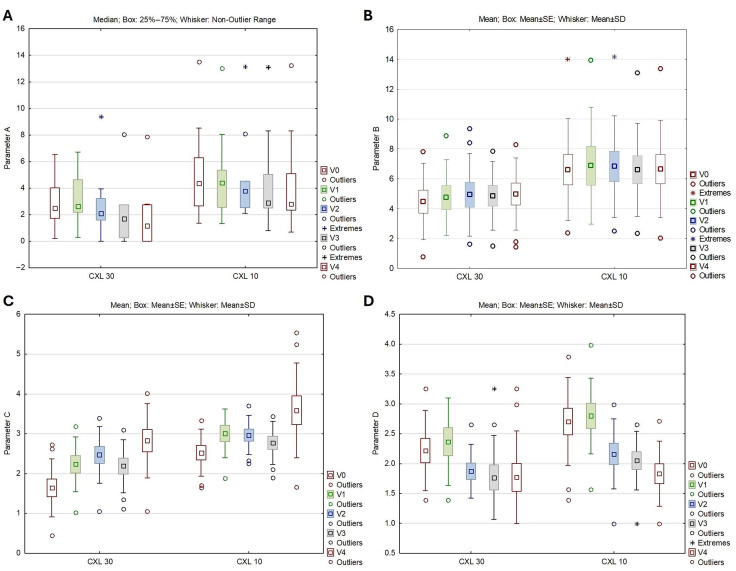
Parameters A (**A**), B (**B**), C (**C**), and D (**D**) in the CXL 30 and CXL 10 groups at baseline (V0) and at 1 month (V1), 3 months (V2), 6 months (V3), and 12 months (V4) after corneal cross-linking.

In the CXL 30 group, all ABCD classification parameters, together with ARC and TP, showed significant changes over time from baseline to follow-up visits (per-protocol set), whereas in the CXL 10 group, significant changes were observed for parameters C and D, as well as ARC (Table 3, Figure 1A–D). By contrast, PRC remained stable throughout follow-up in both groups (Table 3). Post hoc analysis showed that parameter A decreased significantly in the CXL 30 group between V2 and V3 (Z = 2.665, *p* = 0.008), while parameter B increased significantly from V1 to V2 in the same group (*p* = 0.048). Parameter C increased significantly in both groups from V0 to V1 (CXL 30 *p* < 0.001; CXL 10 *p* = 0.027) and again from V3 to V4 (CXL 30 *p* < 0.001; CXL 10 *p* = 0.006). Parameter D decreased significantly between V1 and V2 in both groups (CXL 30 *p* = 0.015; CXL 10 *p* < 0.001). ARC increased significantly from V2 to V3 in both groups (CXL 30 *p* = 0.012; CXL 10 *p* = 0.044), whereas TP showed a significant decrease from V0 to V1 only in the CXL 30 group (*p* < 0.001).

In the CXL 30 group, the remaining analyzed variables (BAD-D, PI, ARTmax, F Km, B Km, PA, and El. F) also showed significant changes across follow-up visits compared with baseline (per-protocol set), whereas in the CXL 10 group, significant change was observed only for ARTmax (Table 4). Post hoc analysis indicated that, in the CXL 30 group, most of these changes occurred between V0 and V1: BAD-D (Z = 2.191, *p* = 0.028) and PI (*p* = 0.037) increased significantly, while ARTmax (*p* = 0.002) and PA (*p* < 0.001) decreased significantly. In addition, F Km (*p* = 0.001) and El. F (*p* = 0.001) decreased significantly from V1 to V3, and B Km (*p* = 0.001) decreased significantly from V1 to V2. In the CXL 10 group, only ARTmax showed a significant decrease from V0 to V1 (*p* = 0.031). El. B remained unchanged throughout follow-up in both groups (*p* > 0.05).

## 4. Discussion

At the later follow-up, most eyes maintained the improvements already seen at 6 months, although some structural and functional parameters continued to change between 6 and 12 months. This suggests that the effects of cross-linking may still evolve during the second half of the first postoperative year, even though the overall picture at 12 months remains one of stabilization.

At one year, visual and keratometric outcomes were broadly similar between the two protocols, while within-group tomographic changes appeared more extensive in the conventional CXL group. However, differences in within-group statistical significance should not be interpreted as definitive evidence of true between-protocol differences. This suggests that, despite similar clinical effectiveness in halting progression, the underlying remodeling response may differ between protocols. One possible explanation is that, despite equal total fluence, differences in irradiance and exposure time may influence the depth and distribution of cross-linking under oxygen-limited conditions. However, this mechanistic interpretation remains speculative in the present study because biomechanical and densitometric endpoints were not assessed.

Earlier comparative studies of CXL protocols focused mainly on K-metrics and anterior corneal parameters, with much less longitudinal information on posterior changes or the ABCD system [21,22]. More recently, ABCD outcomes have begun to appear in studies with approximately 1-year follow-up, including multi-arm analyses showing protocol-related differences in A and B alongside keratometric changes [19,29,30]. For example, Danesh et al. reported one-year ABCD changes after conventional CXL and illustrated how this staging approach can capture posterior corneal behavior and visual function together with structural change [19]. Similarly, the previously published 6-month head-to-head analysis of this cohort comparing conventional CXL with the 9 mW/10 min accelerated protocol highlighted how much of the earlier literature had relied mainly on K-metrics and anterior tomography, while also suggesting a more pronounced anterior tomographic response after conventional treatment despite similar short-term clinical efficacy [28].

A randomized head-to-head trial by Hagem et al. also reported comparable 2-year outcomes between conventional and accelerated epi-off CXL performed with HPMC-based riboflavin, supporting the view that both protocols can achieve similar short- to mid-term clinical stabilization [31]. Studies that examined ABCD by cone location, such as Krolo et al., suggest that the apparent effect size may depend in part on cone geometry, which is one reason ABCD can add information beyond Kmax alone [20]. Although we did not stratify our cohort by cone location, our one-year findings also pointed mainly toward anterior remodeling, while posterior changes were less pronounced overall [20,30]. Meta-analyses by Shajari et al. and by Kobashi and Tsubota likewise indicate that most head-to-head comparisons have historically focused on anterior or keratometric endpoints [21,22]. Overall, although ABCD has appeared more frequently in studies with about 1 year of follow-up, direct 12-month head-to-head comparisons between conventional and accelerated epi-off CXL, using ABCD as the main comparator, still appear unavailable.

The previously published 6-month analysis of this cohort showed similar functional improvement in both groups, but earlier and more pronounced anterior changes in the CXL 30 group [28]. After extending follow-up to 12 months, we again observed overall stabilization, together with a reduction in A stage, an increase in ARC, and a decrease in Kmax, all of which suggest a more pronounced anterior tomographic response after conventional CXL in within-group analyses. Posterior tomographic findings were less consistent than anterior findings. PRC and posterior elevation remained largely stable, whereas the ABCD B parameter changed significantly over time in the conventional group. Because this B-stage change was not accompanied by significant changes in raw PRC or posterior elevation, it should be interpreted as an exploratory posterior tomographic signal rather than definitive evidence of clinically meaningful posterior remodeling. Overall, these findings suggest that both protocols achieved clinical stabilization within 1 year, whereas the conventional CXL group exhibited a broader pattern of anterior tomographic change in this exploratory cohort. This observation should be interpreted with caution given the small sample size, baseline imbalance, and the primarily within-group nature of several analyses.

Anterior remodeling was the dominant structural finding of this study. Two parallel changes were evident: A stage improved, and ARC increased, indicating flattening of the anterior corneal surface. Both protocols showed this general pattern, but it was more pronounced and more consistently observed in the conventional CXL group. Kmax also decreased in both groups, whereas front mean keratometry and anterior elevation decreased only in CXL 30. Taken together, these findings suggest a more pronounced anterior flattening pattern in the conventional group; however, this should be interpreted as an exploratory tomographic signal rather than definitive evidence of protocol superiority. Although some anterior changes continued beyond 6 months, the magnitude of additional change in the later interval was smaller, suggesting gradual stabilization of the remodeling process.

It is important to distinguish among structural remodeling, measurement variability, and clinically meaningful control of ectasia. In this small exploratory cohort, isolated statistically significant changes in secondary tomographic indices may reflect postoperative fluctuation, measurement variability, or reference-surface recalculation rather than clinically relevant protocol-specific effects. By contrast, clinically meaningful disease control was supported primarily by stabilization or improvement in Kmax and visual acuity, together with the absence of tomographic signs suggesting further posterior protrusion. Therefore, the overall pattern of change, consistency across related parameters, and functional stability were considered more informative than individual *p*-values alone.

Posterior tomographic changes were limited and less consistent than anterior changes. PRC and posterior elevation remained largely stable in both groups during follow-up. Although the ABCD B parameter and mean back keratometry (B Km) changed significantly in the conventional CXL group, these findings were not accompanied by significant changes in raw PRC or posterior elevation. Therefore, they should be interpreted with caution as exploratory posterior tomographic signals rather than as definitive evidence of clinically meaningful posterior remodeling. The stability of posterior elevation between 6 and 12 months argues against further posterior protrusion and supports continued disease control. Overall, late remodeling was predominantly anterior.

Changes in pachymetric and relational indices followed the expected postoperative pattern. Parameter C primarily reflected postoperative pachymetric behavior and should not be interpreted in isolation as evidence of ectatic progression or regression. In this cohort, C reflected early postoperative thinning followed by partial recovery, while TP and PA showed an early decrease, more clearly in CXL 30, and then stabilized. ARTmax decreased in both groups, likely due to early pachymetric and gradient changes, and then remained relatively stable. PI increased early in CXL 30 and later partially regressed or stabilized, whereas only minimal change was observed in CXL 10. Taken together with the increase in ARC, the decrease in Kmax, and the stability of PRC, these findings are more consistent with procedure-related pachymetric effects than with ongoing ectatic progression.

Early changes in BAD-D may likewise reflect postoperative corneal remodeling and recalculation of the reference surface. Although the conventional CXL group showed a broader pattern of anterior tomographic change, both protocols were associated with functional improvement and Kmax stabilization at one year. Therefore, the observed tomographic differences should not be interpreted as evidence of clinically relevant superiority of one protocol over the other. Rather, they may indicate protocol-related differences in structural response that require confirmation in larger, adequately powered studies. UDVA and BCVA improved mainly during the first 3 to 6 postoperative months and then remained relatively stable. These findings support the 12-month visit as a useful time point for reassessing stability after CXL, but they do not define optimal timing for retreatment or optical rehabilitation.

Although a 12-month follow-up provides clinically useful information on early stabilization and remodeling after CXL, it remains insufficient for assessing the long-term durability of the treatment effect. Keratoconus progression may recur several years after apparent early stabilization, and late biomechanical or tomographic changes cannot be excluded on the basis of one-year data alone. Longer follow-up is therefore required to determine whether the observed stabilization and anterior tomographic response are sustained over time.

This study has several limitations. The most important limitation is the small sample size, which limits statistical power, reduces the precision of estimates, and restricts the ability to detect definitive between-group differences across multiple tomographic parameters. No formal a priori sample size calculation was performed because this was an exploratory continuation study of a previously reported cohort. Therefore, non-significant findings should not be interpreted as evidence of equivalence between protocols, and the between-group comparisons should be regarded as exploratory. Multiple repeated comparisons were performed across several tomographic and functional parameters. Because formal multiplicity correction was not applied across all analyses, the risk of type I error inflation should be considered, and isolated statistically significant secondary findings should be interpreted with caution. The study was also conducted at a single center and did not involve randomization. Because treatment allocation was based on clinical judgment rather than randomization, selection bias and confounding by indication cannot be excluded. In particular, the significant baseline differences in age and sex between the two groups may have influenced postoperative remodeling patterns and limit causal interpretation of between-protocol comparisons. These imbalances should therefore be considered when assessing the robustness and interpretability of the comparative findings. In addition, both eyes of two participants were included in the analysis, which may introduce inter-eye correlation and affect variance estimates. Nevertheless, the small number of bilateral cases and the limited sample size should be considered when interpreting statistical significance. The analysis was performed on a per-protocol basis, which may introduce attrition bias due to the exclusion of patients who did not complete follow-up. We also did not assess densitometry or stratify by cone location, both of which may provide additional mechanistic insight in future studies. Finally, Scheimpflug tomography is subject to inherent measurement variability.

## 5. Conclusions

At one year, both conventional (CXL 30) and accelerated (CXL 10) epi-off cross-linking were associated with stabilization of progressive keratoconus and improved visual function. Corneal remodeling beyond 6 months was predominantly anterior. Within-group findings suggested a more pronounced anterior tomographic response in the conventional group; however, this observation remains exploratory and should be interpreted in light of the small sample size, baseline imbalance, and non-randomized design. Posterior metrics were largely stable, and isolated changes in posterior-related indices should be interpreted cautiously. The ABCD framework, when used alongside keratometry, may capture multidimensional corneal changes more comprehensively than apical K-metrics alone.

Clinically, the 12-month visit may be a useful time point for reassessing stability after CXL, although this study was not designed to determine optimal retreatment timing or optical rehabilitation strategy. Larger randomized or well-adjusted prospective studies with longer follow-up are warranted to confirm these findings and determine whether the observed tomographic differences translate into clinically meaningful advantages.

## Figures and Tables

**Table 1:** Baseline characteristics of the 20 patients (22 eyes) included in the study, stratified according to the CXL protocol.

	CXL 30 (9 Patients; 11 Eyes)	CXL 10 (11 Patients; 11 Eyes)	t ^a^ χ^2 b^ Z^c^	*p*
Age (years) *	22.11 ± 4.34	28.09 ± 5.03	−3.037 ^a^	**<0.001 ** ^a^
Gender (m/f) **	6/3	11/0	4.314 ^b^	**0.038** ^b^
Eye (right/left) **	7/4	4/7	0.636 ^b^	0.201 ^b^
Kmax ≥ 55, BCVA ≤ 0.5/Kmax < 55, BCVA > 0.5 **	8/3	11/0	2.888 ^b^	0.089 ^b^
Kmax †	56.5 (47, 77)	59.3 (51, 90)	−1.609 ^c^	0.108 ^c^
UDVA (decimal) †	0.2 (0.05, 0.8)	0.2 (0.01, 0.6)	0.591 ^c^	0.554 ^c^
Sphere (dptr) †	−1.00 (−8.00, 0.00)	−0.75 (−6.00, 0.50)	0.131 ^c^	0.896 ^c^
Cylinder (dptr) †	−1.25 (−3.50, 0.00)	−2.50 (−5.00, 0.00)	1.609 ^c^	0.108 ^c^
BCVA (decimal) †	0.4 (0.15, 0.8)	0.2 (0.08, 0.8)	1.445 ^c^	0.149 ^c^

* mean ± SD ** number † median (min, max) ^a^ *t*-test ^b^ Chi-square test df = 1 ^c^ Mann–Whitney test, Kmax-maximal keratometry, UDVA-uncorrected distance visual acuity, BCVA-best corrected visual acuity. Bold *p*-values indicate statistically significant differences at *p* < 0.05.

**Table 2:** Visual acuity and maximal keratometry in the CXL 30 and CXL 10 groups at baseline and at 1, 3, 6, and 12 months following corneal cross-linking.

**UDVA**							
	V0	V1	V2	V3	V4	χ^2^	*p*
CXL 30	0.2 (0.1, 0.8)	0.3 (0.1, 0.7)	0.5 (0.1, 0.7)	0.5 (0.1, 1.0)	0.6 (0.1, 1.0)	12.816	**0.012**
CXL 10	0.2 (0.1, 0.6)	0.2 (0.1, 0.6)	0.3 (0.1, 0.6)	0.3 (0.1, 0.6)	0.3 (0.1, 0.6)	11.573	**0.021**
**BCVA**							
	V0	V1	V2	V3	V4	χ^2^	*p*
CXL 30	0.4 (0.2, 0.8)	0.4 (0.2, 0.8)	0.6 (0.3, 0.8)	0.7 (0.2, 1.0)	0.8 (0.2, 1.0)	23.836	**<0.001**
CXL 10	0.2 (0.1, 0.8)	0.3 (0.1, 0.7)	0.4 (0.2, 1.0)	0.5 (0.3, 1.0)	0.5 (0.3, 1.0)	30.490	**<0.001**
**Kmax**							
	V0	V1	V2	V3	V4	χ^2^	*p*
CXL 30	56.5 (47, 77)	57.3 (48, 80)	55.1 (46, 76)	53.1 (46, 76)	52.3 (45, 73)	29.653	**<0.001**
CXL 10	59.3 (51, 90)	60.5 (51, 87)	59.4 (52, 87)	59.8 (50, 88)	58.3 (51, 90)	11.096	**0.026**

Median (min, max) Friedman ANOVA test. UDVA-uncorrected distance visual acuity (decimal), BCVA-best corrected visual acuity (decimal), Kmax-maximal keratometry, V0-baseline examination, V1-one month postoperatively, V2-three months postoperatively, V3-six months postoperatively, V4-one year postoperatively. Bold *p*-values indicate statistically significant differences at *p* < 0.05.

**Table 3:** ABCD grading system parameters, ARC, PRC, and TP in the CXL 30 and CXL 10 groups at baseline and at 1, 3, 6, and 12 months after corneal cross-linking.

Parameter A *							
Group	V0	V1	V2	V3	V4	χ^2^	*p*
CXL 30	2.5 (0.2, 6.5)	2.6 (0.3, 6.7)	2.1 (0, 9.4)	1.7 (0, 8.1)	1.2 (0, 7.9)	18.276	**0.001 ** ^a^
CXL 10	4.4 (1.4, 13)	4.4 (1.3, 13)	3.8 (2.1, 13)	2.9 (0.8, 13)	2.8 (0.7, 13)	7.573	0.108 ^a^
Parameter B **							
Group	V0	V1	V2	V3	V4	F	*p*
CXL 30	4.47 ± 2.55	4.74 ± 2.54	4.93 ± 2.77	4.85 ± 2.29	4.98 ± 2.41	2.682	**0.047** ^b^
CXL 10	6.61 ± 3.41	6.88 ± 3.91	6.82 ± 3.40	6.61 ± 3.12	6.65 ± 3.24	0.415	0.797 ^b^
**Parameter C** **							
Group	V0	V1	V2	V3	V4	F	*p*
CXL 30	1.64 ± 0.73	2.23 ± 0.69	2.47 ± 0.71	2.19 ± 0.67	2.82 ± 0.93	23.853	**<0.001** ^b^
CXL 10	2.53 ± 0.59	3.01 ± 0.62	2.97 ± 0.49	2.77 ± 0.54	3.59 ± 1.19	7.460	**<0.001** ^b^
Parameter D **							
Group	V0	V1	V2	V3	V4	F	*p*
CXL 30	2.22 ± 0.67	2.37 ± 0.73	1.87 ± 0.45	1.77 ± 0.70	1.77 ± 0.78	9.042	**<0.001** ^b^
CXL 10	2.71 ± 0.74	2.80 ± 0.63	2.16 ± 0.59	2.05 ± 0.49	1.83 ± 0.55	19.202	**<0.001** ^b^
ARC **							
Group	V0	V1	V2	V3	V4	F	*p*
CXL 30	6.65 ± 0.60	6.59 ± 0.60	6.78 ± 0.79	6.96 ± 0.73	6.99 ± 0.75	8.105	**<0.001** ^b^
CXL 10	6.03 ± 0.80	6.05 ± 0.84	6.12 ± 0.75	6.19 ± 0.83	6.23 ± 0.86	3.765	**0.013** ^b^
PRC **							
Group	V0	V1	V2	V3	V4	F	*p*
CXL 30	4.95 ± 0.63	4.88 ± 0.61	4.85 ± 0.66	4.66 ± 0.96	4.81 ± 0.57	2.009	0.114 ^b^
CXL 10	4.12 ± 0.96	3.99 ± 1.06	4.14 ± 0.80	4.12 ± 0.92	4.09 ± 0.99	0.565	0.690 ^b^
TP **							
Group	V0	V1	V2	V3	V4	F	*p*
CXL 30	464.32 ± 34.54	435.33 ± 33.31	422.91 ± 36.95	437.74 ± 31.32	440.31 ± 31.46	13.263	**<0.001** ^b^
CXL 10	425.61 ± 34.87	405.54 ± 60.51	399.76 ± 38.61	412.01 ± 35.52	414.43 ± 41.63	2.634	0.052 ^b^

* median (min, max) ** mean ± SD ^a^ Friedman ANOVA test ^b^ Repeated measures ANOVA test. ARC, anterior radius of curvature measured within a 3.0 mm optical zone centered on the thinnest corneal point; PRC, posterior radius of curvature measured within a 3.0 mm optical zone centered on the thinnest corneal point; TP, thinnest pachymetry; V0, baseline visit; V1, 1 month postoperatively; V2, 3 months postoperatively; V3, 6 months postoperatively; V4, 12 months postoperatively. Bold *p*-values indicate statistically significant differences at *p* < 0.05.

**Table 4:** Corneal parameters and progression indices in the CXL 30 and CXL 10 groups at baseline and at 1, 3, 6, and 12 months after corneal cross-linking. Bold *p*-values indicate statistically significant differences at *p* < 0.05.

**BAD-D**							
Group	V0	V1	V2	V3	V4	χ^2^	*p*
CXL 30	7.3 (1.5, 16)	9.9 (1.6, 19)	9.2 (2.2, 21)	8.1 (4.1, 18.3)	9.6 (4.1, 18)	18.020	**0.001** ^a^
CXL 10	12.7 (6.4, 34)	13.2 (6.4, 34)	13.5 (6.7, 33)	12.8 (6.1, 32)	13.1 (4.9, 31)	7.822	0.098 ^a^
**PI**							
Group	V0	V1	V2	V3	V4	F	*p*
CXL 30	2.15 ± 0.70	2.56 ± 0.85	2.84 ± 0.94	2.81 ± 0.73	2.80 ± 0.73	5.219	**0.002** ^b^
CXL 10	2.75 ± 0.96	3.46 ± 1.76	3.30 ± 1.35	3.03 ± 0.91	3.08 ± 1.15	2.570	0.057 ^b^
**ARTmax**							
Group	V0	V1	V2	V3	V4	F	*p*
CXL 30	157.73 ± 59.25	115.50 ± 46.68	117.36 ± 42.56	126.45 ± 44.63	124.45 ± 40.63	4.701	**0.003 ^b^**
CXL 10	117.73 ± 49.16	96.44 ± 53.15	96.45 ± 38.85	104.00 ± 39.48	111.73 ± 47.64	4.730	**0.004** ^b^
**F Km**							
Group	V0	V1	V2	V3	V4	F	*p*
CXL 30	48.29 ± 3.93	49.09 ± 3.93	47.55 ± 4.56	46.72 ± 4.14	46.69 ± 4.19	19.154	**<0.001** ^b^
CXL 10	47.41 ± 16.85	52.91 ± 7.75	51.60 ± 7.10	51.20 ± 7.60	50.68 ± 7.49	0.827	0.518 ^b^
**B Km**							
Group	V0	V1	V2	V3	V4	F	*p*
CXL 30	−7.25 ± 0.78	−7.13 ± 0.82	−7.32 ± 0.80	−7.31 ± 0.76	−7.34 ± 0.75	9.890	**<0.001** ^b^
CXL 10	−7.89 ± 1.43	−8.14 ± 1.65	−8.00 ± 1.50	−7.88 ± 1.33	−7.85 ± 1.36	1.216	0.323 ^b^
**PA**							
Group	V0	V1	V2	V3	V4	F	*p*
CXL 30	475.27 ± 31.93	449.30 ± 28.74	441.91 ± 29.88	451.18 ± 25.78	453.82 ± 23.97	16.803	**<0.001** ^b^
CXL 10	438.73 ± 35.15	403.33 ± 47.04	412.27 ± 39.84	381.25 ± 128.95	426.00 ± 43.56	1.175	0.340 ^b^
**El. F**							
Group	V0	V1	V2	V3	V4	F	*p*
CXL 30	22.82 ± 11.24	25.70 ± 9.73	22.82 ± 16.70	19.09 ± 17.17	17.36 ± 14.53	2.681	**0.047** ^b^
CXL 10	36.73 ± 19.63	36.00 ± 21.06	34.55 ± 18.23	32.82 ± 20.50	31.55 ± 21.35	1.984	0.121 ^b^
**El. B**							
Group	V0	V1	V2	V3	V4	F	*p*
CXL 30	52.09 ± 26.12	57.70 ± 24.00	55.73 ± 27.83	57.73 ± 26.71	57.82 ± 24.21	2.299	0.077 ^b^
CXL 10	76.45 ± 38.71	78.33 ± 44.64	78.09 ± 39.61	74.73 ± 37.29	74.00 ± 38.01	0.494	0.740 ^b^

Data are presented as median (minimum, maximum) or mean ± standard deviation (SD), as applicable. Bold *p*-values indicate statistically significant differences at *p* < 0.05. ^a^ Friedman ANOVA test ^b^ Repeated measures ANOVA test. BAD-D, Belin/Ambrósio enhanced ectasia total deviation; PI, pachymetric progression index; ARTmax, maximum Ambrosio relational thickness; F Km, mean front keratometry; B Km, mean back keratometry; PA, pachymetry at the apex; El. F, anterior corneal elevation; El. B, posterior corneal elevation; V0, baseline visit; V1, 1 month postoperatively; V2, 3 months postoperatively; V3, 6 months postoperatively; V4, 12 months postoperatively.

## Data Availability

Individual participant data are not publicly available due to privacy and consent restrictions. De-identified aggregate data (summary tables) may be obtained from the corresponding author upon reasonable request, subject to institutional approval.

## Abbreviations

The following abbreviations are used in this manuscript:

ABCD	Belin ABCD keratoconus classification (A, anterior curvature stage; B, posterior curvature stage; C, thinnest pachymetry stage; D, distance BCVA stage), stages 0–4
CXL 10	Accelerated epi-off cross-linking (9 mW/cm^2^ × 10 min; ~5.4 J/cm^2^)
ARTmax	Ambrosio Relational Thickness (maximum)
BCVA	Best-corrected visual acuity (decimal Snellen)
B(PRC stage)	Posterior curvature stage (ABCD, 0–4)
BAD-D	Belin/Ambrósio deviation (composite ectasia index)
B Km	Mean back keratometry (diopters)
C(TP stage)	Thinnest pachymetry stage (ABCD, 0–4)
CXL	Corneal collagen cross-linking
CXL 30	Conventional (Dresden) epi-off cross-linking (3 mW/cm^2^ × 30 min; ~5.4 J/cm^2^)
D(BCVA stage)	Distance visual acuity stage (ABCD, 0–4; lower = better)
DL	Demarcation line (post-CXL stromal interface on AS-OCT/confocal)
El. B	Posterior elevation at the thinnest point (relative to best-fit reference surface)
El. F	Anterior elevation at the thinnest point (relative to best-fit reference surface)
Epi-off	Epithelium-off technique
F Km	Mean front keratometry (diopters)
IQR	Interquartile range
Kmax	Maximum anterior keratometry (diopters)
PA	Pachymetry at the apex (µm)
PI	Pachymetry/ectasia “progression indices” (Pentacam).
PRC	Posterior radius of curvature at the thinnest point (mm); higher = flatter
SD	Standard deviation
TP	Thinnest pachymetry (µm)
UDVA	Uncorrected distance visual acuity (decimal Snellen)
UVA	Ultraviolet-A irradiation–
V0–V4	Study visits: V0 baseline; V1 1 month; V2 3 months; V3 6 months; V4 12 months
